# The Impact of Cesarean Section Delivery on Intestinal Microbiota: Mechanisms, Consequences, and Perspectives—A Systematic Review

**DOI:** 10.3390/ijms25021055

**Published:** 2024-01-15

**Authors:** Francesco Inchingolo, Alessio Danilo Inchingolo, Irene Palumbo, Irma Trilli, Mariafrancesca Guglielmo, Antonio Mancini, Andrea Palermo, Angelo Michele Inchingolo, Gianna Dipalma

**Affiliations:** 1 Department of Interdisciplinary Medicine, School of Medicine, University of Bari “Aldo Moro”, 70124 Bari, Italy; ad.inchingolo@libero.it (A.D.I.); irenepalu@icloud.com (I.P.); trilliirma@gmail.com (I.T.); m.guglielmo2@studenti.uniba.it (M.G.); dr.antonio.mancini@gmail.com (A.M.); angeloinchingolo@gmail.com (A.M.I.); giannadipalma@tiscali.it (G.D.); 2 College of Medicine and Dentistry, Birmingham B4 6BN, UK; andrea.palermo2004@libero.it

**Keywords:** neonatal bacterial assemblages, infant gut microbiota, caesarean delivery, Bacteroides, delivery mode, transmission of maternal strains, infant oral microbiota

## Abstract

The relationship between cesarean section (CS) delivery and intestinal microbiota is increasingly studied. CS-born infants display distinct gut microbial compositions due to the absence of maternal birth canal microorganisms. These alterations potentially link to long-term health implications like immune-related disorders and allergies. This correlation underscores the intricate connection between birth mode and the establishment of diverse intestinal microbiota. A systematic literature review was conducted on the PubMed, Scopus, and Web of Science databases by analyzing the articles and examining the intricate interactions between CS delivery and the infant’s intestinal microbiota. The analysis, based on a wide-ranging selection of studies, elucidates the multifaceted dynamics involved in CS-associated shifts in the establishment of fetal microbiota. We also explore the potential ramifications of these microbial changes on neonatal health and development, providing a comprehensive overview for clinicians and researchers. By synthesizing current findings, this review contributes to a deeper understanding of the interplay between delivery mode and early microbial colonization, paving the way for informed clinical decisions and future investigations in the field of perinatal medicine.

## 1. Introduction

The intricate interplay between the human gut microbiota (GM) and overall health has ignited a burgeoning interest in understanding the role of birth mode, particularly cesarean section (CS) delivery, in shaping the composition and development of the infant’s intestinal microbial community [1,2,3,4,5,6].

The microbiota, encompassing a vast array of microorganisms such as bacteria, viruses, fungi, and archea, plays an indispensable role in numerous physiological processes, ranging from nutrient absorption to immune system modulation [7,8,9,10,11,12,13,14,15]. It is believed that archaea may actively participate in the metabolism of compounds in the gut, thus influencing the overall composition of the microbial community. Furthermore, some studies suggest that archaea may play a key role in the degradation of complex compounds, thereby contributing to the production of substances that could have systemic effects beyond the intestinal environment [16].

Consequently, the mode of birth, with its potential to influence the early colonization of the infant’s gut, has become a focal point of scientific inquiry [17,18,19,20,21,22].

### 1.1. Context and Significance

In an era marked by rapid advancements in medical technology, the prevalence of CS deliveries has witnessed a remarkable escalation [23,24,25,26,27,28,29,30,31].

Although CSs are often performed for medical reasons, including cases involving maternal health, fetal distress, or malpresentation, there has been a growing concern about the potential consequences of this surgical procedure on the neonate’s microbiota [2,32,33,34,35,36,37].

In various regions worldwide, CS rates have risen to unprecedented levels, leading researchers to scrutinize the potential ramifications of this trend on infant health. Consequently, understanding the implications of CS delivery on the infant’s GM holds profound significance for maternal and child health outcomes [38,39,40,41,42,43,44,45,46,47,48,49,50,51].

### 1.2. Mechanisms Involved

The mechanisms underpinning the impact of CS delivery on the infant’s GM are multifaceted [41,52,53,54,55].

One of the most significant differences between CS and vaginal delivery lies in the initial microbial exposure of the neonate [56,57].

During a vaginal birth, neonates traverse the birth canal, encountering a diverse array of maternal microorganisms that confer an early inoculation of the infant’s gut [58,59,60].

These maternal microbes, ranging from lactobacilli to bifidobacteria, provide a foundation for a healthy microbial community within the neonate [61,62].

In contrast, CS-born neonates miss this crucial exposure as they bypass the birth canal and are instead exposed to environmental microbes prevalent in the hospital setting and on the maternal skin. This microbial incongruence can potentially shape the neonate’s GM composition and diversity in distinct ways [3,63,64,65,66].

### 1.3. Long-Term Health Implications

The importance of early microbial colonization in shaping long-term health outcomes cannot be overstated [1,9,67,68,69,70].

The composition of the infant’s GM during the first critical months of life is believed to exert a lasting influence on the individual’s health trajectory [18,21,34,71].

Emerging evidence suggests that deviations from the natural process of vaginal birth, such as through CS delivery, can contribute to alterations in the microbiota that may have far-reaching consequences [20,72,73].

Disruptions in the establishment of a balanced and diverse microbiota composition have been implicated in various health conditions, including autoimmune disorders, metabolic syndrome, and even mental health disorders [18,74,75,76,77,78].

These findings underscore the significance of investigating the potential role of CS delivery in contributing to such health outcomes [25,36,79,80].

### 1.4. Study Objectives

Considering the growing concern surrounding the impact of CS delivery on the infant’s GM, the primary objective of this article is to comprehensively dissect and analyze the intricate relationship between birth mode and the intestinal microbial ecosystem. This investigation will encompass an exploration of the differences in microbial community structure, diversity, and the functional potential between CS and vaginal births [81]. Moreover, by delving into the underlying mechanisms behind these differences, such as microbial exposure and maternal–fetal interactions, this study aims to shed light on the intricate processes governing the establishment of the infant’s GM in the context of CS deliveries [82].

**Figure 1:**
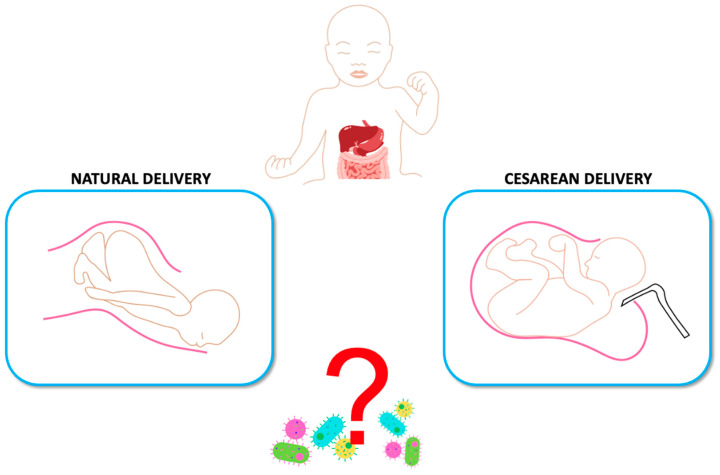
Graphic illustration of the purpose of this systematic review, differences between the infant microbiota in natural or cesarean delivery.

In summary, this article serves as a comprehensive exploration of the intricate interactions between CS delivery and the infant’s intestinal microbiota (Figure 1). By examining the mechanisms, consequences, and long-term implications of CS-related microbial alterations, this study contributes to a deeper understanding of the multifaceted relationship between birth mode and microbial colonization. This understanding, in turn, could pave the way for informed decisions and potential interventions that support the establishment of a healthy GM in infants born through CS.

## 2. Methods

### 2.1. Protocol and Registration

This systematic review was conducted according to Preferred reporting items for systematic reviews and meta-analyses (PRISMA) and the protocol was registered at PROSPERO under the ID of CDR 469789.

### 2.2. Search Processing

To locate studies that matched the topic of the influence of precision medicine and oral health, a search was conducted on PubMed, Scopus, and Web of Science for papers published between 1 January 2013 and 1 July 2023. The search strategy used the Boolean keywords of “cesarean delivery” AND (“infant gut microbiota” OR “infant oral microbiota”) (Table 1).

### 2.3. Inclusion Criteria

The following inclusion criteria were considered: (1) open-access studies; (2) studies that investigated the relationship between the influence of CS delivery and the infant’s GM; (3) randomized clinical trials, comprising retrospective and observational studies; (4) use of the English language; and (5) full-text.

Papers that did not match the above criteria were excluded; the review was conducted using the PICOS criteria as follows:

- Participants: infant patients, both male and female;
- Interventions: applications of C- section delivery;
- Comparisons: infant administration of vaginal microbiota;
- Outcomes: infant’s GM during the first 1000 days of life is critical for preventing various health issues in later life;
- Study: randomized clinical trials, retrospective and observational studies.

### 2.4. Exclusion Criteria

The exclusion criteria were as follows: (1) animal studies; (2) in vitro studies; (3) off-topic studies; (4) reviews, case reports, case series, letters, or comments; (5) no use of the English language.

### 2.5. Data Processing

Based on selection criteria, three reviewers (M.G., I.P., and I.T.) independently accessed the databases to gather the studies and assigned a quality rating. Zotero (v6.0.15) was used to download the chosen articles. Disagreements amongst the three writers were resolved through consultation with a senior reviewer (F.I.).

### 2.6. Quality Assessment

The quality of the included papers was assessed by two reviewers, RF and EI, using the ROBINS-I tool developed to assess risk of bias in the results of non-randomized studies that compare the health effects of two or more interventions. Seven points were evaluated and each was assigned a degree of bias. A third reviewer (FI) was consulted in the event of a disagreement until an agreement was reached.

## 3. Results and Discussion

### 3.1. Study Selection and Characteristics

The electronic database search identified a total of 484 articles (Scopus *n* = 24, PubMed *n* = 226, Web of Science *n* = 234), and no articles were included through the hand search.

**Figure 2:**
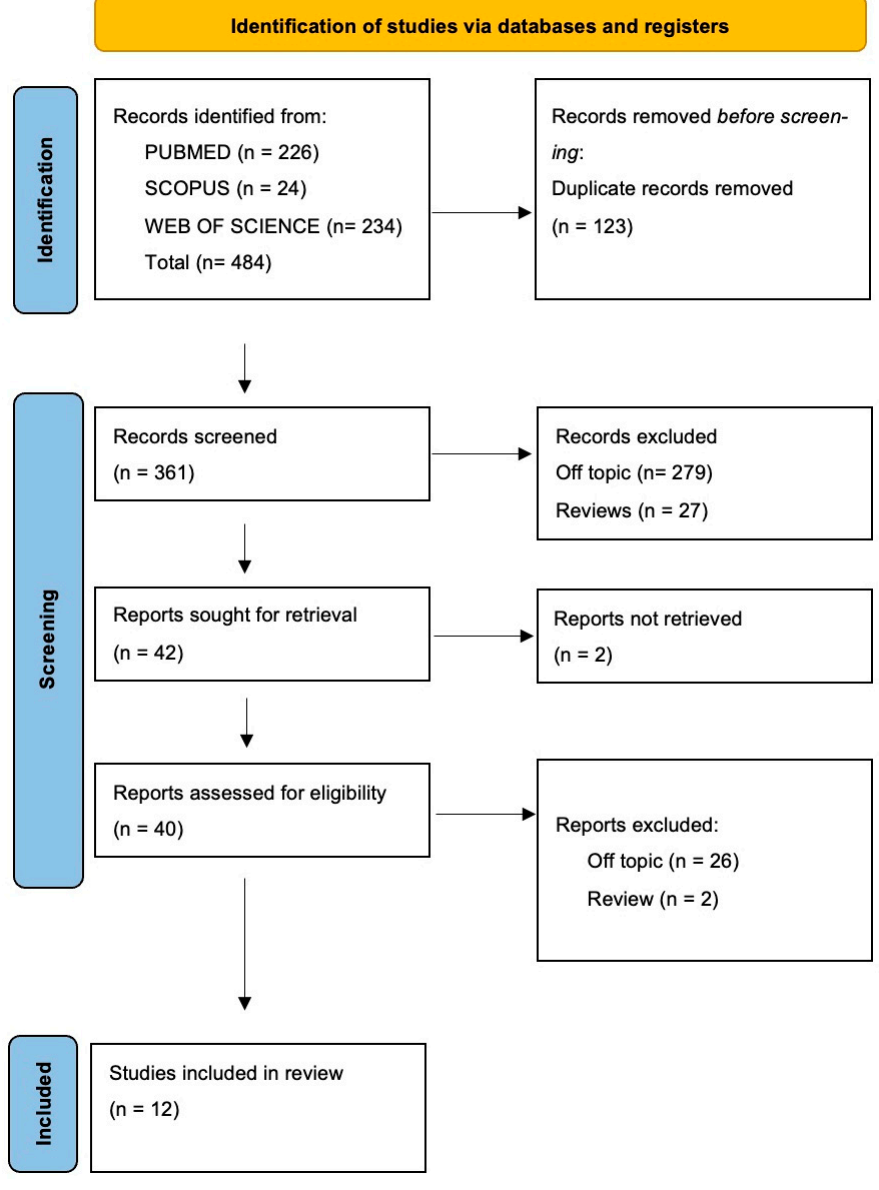
Flow diagram and database search indicators of the preferred search of the literature reporting items for systematic reviews and meta-analyses (PRISMA).

After the deletion of duplicates, 361 studies were screened by evaluating the title and abstract, focusing on the association between precision medicine, genomics, and their implications in oral health. There were 319 articles that did not meet the inclusion criteria (279 off-topic, 27 review, 13 in vitro studies), thus leading to 42 records being selected. Subsequently, 2 records that were non-retrieved were excluded, and then 28 reports were excluded because they did not meet the inclusion criteria (26 off-topic, 2 review). After eligibility, 10 records were selected for qualitative analysis. The selection process and the summary of selected records are shown in Figure 2 and Table 2, respectively.

### 3.2. Quality Assessment and Risk of Bias

**Figure 3:**
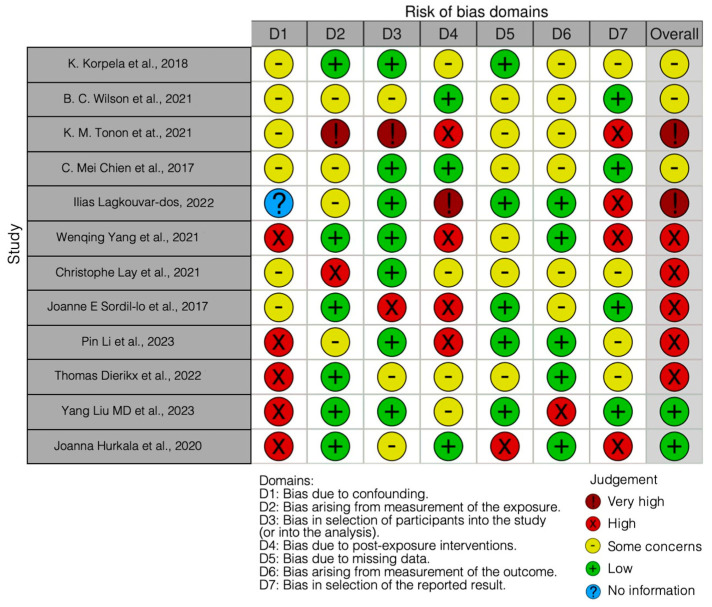
Bias of the included studies using the Robins Tool [10,69,72,83,84,85,86,87,88,89,90,91].

The risk of bias in the included studies is reported in Figure 3. Bias resulting from confounding the majority of studies is a high risk form of bias, while that arising from measurement parameter is a low risk form of bias. Many studies have a low risk of bias due to bias in the selection of participants. Bias due to post-exposure cannot be calculated due to high heterogeneity. Bias due to missing data is low in many studies. Bias arising from measurement of the outcome is low. Bias in the selection of the reported results is high in most studies. The final results show that five studies have a high risk of bias, two have a very high risk of bias, and two have a low risk of bias.

Over time, it has become increasingly evident that the mode of birth, whether it be cesarean or natural, exerts a profound influence on the composition of an infant’s GM. A study conducted by K. Korpela et al. [83] offered valuable insights into this matter. Their comprehensive analysis of the GM in infants unveiled a substantial disparity between those born through cesarean delivery and those born naturally. Specifically, infants delivered through CS exhibited a marked decrease in beneficial bacteria such as Bifidobacterium and Bacteroides, which are pivotal to carbohydrate digestion and bolstering the immune system. This alteration has been linked to a diminished capacity of the microbiota to metabolize carbohydrates, including oligosaccharides found in breast milk [92]. Notably, intervention with a probiotic supplement comprising selected strains of Bifidobacterium and Lactobacillus has been shown to counteract this unfavorable trend. This is particularly significant for infants born through CS and exposed to antibiotics, as probiotic supplementation has demonstrated its ability to restore a more balanced and functional microbiota, averting the loss of bifidobacteria and normalizing crucial microbial functions. These findings underscore the pivotal role of early GM in children’s health and open avenues for potential interventions for mitigating the adverse effects of factors such as cesarean delivery and antibiotic usage on long-term health [83].

Adding a valuable perspective, in the research conducted by B. C. Wilson et al. [84], a pilot trial was carried out to explore the potential impact of a practice known as “vaginal seeding” on the gut microbiome of infants born through CS in comparison with those born vaginally. The primary objective was to investigate whether the oral administration of maternal vaginal microbiota could reestablish the gut microbiome of cesarean-born infants in order to resemble that of vaginally born infants. Regrettably, the results indicated that vaginal seeding did not exert a significant influence on the structure or function of the gut microbiome in cesarean-born infants at both 1 month and 3 months of age. Despite rigorous measures to minimize infection risks through maternal pathogen screening, the procedure did not appear to effectively restore levels of Bacteroides in the infants’ gut microbiomes, a characteristic feature of vaginally born infants. This study also illuminated the challenges and constraints of microbiota-based interventions and suggested that alternative approaches, such as the delayed administration of intrapartum antibiotic prophylaxis or probiotic formulations, might offer safer and more inclusive options for microbiome restoration [93]. These findings imply that the utility of vaginal seeding in reducing disease risks in cesarean-born infants may be limited, prompting further exploration of alternative strategies to promote healthy microbiome development [84].

The research conducted by Yang Liu MD et al. [10] did not detect significant differences in BMI or allergy-related risks between the two groups under investigation. While a lower rate of overweight/obesity was observed in the “vaginal seeding” group at 6 months, the researchers cautioned against overinterpreting this result due to the possibility of type I errors stemming from multiple comparisons. Based on its findings, the study’s conclusion is consequently that “vaginal seeding” does not find support as a practice in clinical settings for infants born at term through cesarean delivery. The researchers advocate for safer alternatives, such as breastfeeding, judicious antibiotic use, and probiotic supplementation. Moreover, the study acknowledges certain limitations, including the absence of bacterial detection in the maternal vagina and gauze, missing data attributed to the COVID-19 pandemic, and the necessity for further investigation into alternative strategies to mimic microbiota exposure during vaginal delivery.

K. M. Tonon et al. [85] delved into the distinctions of the GM in infants born through CS versus natural birth, with a specific emphasis on maternal secretory status and the composition of her human milk oligosaccharides (HMOs). The researchers determined maternal secretory status based on the presence of α1-2 fucosylated structures in the breast milk sample through mass spectrometry (LC-MS). While the overall GM composition and alpha and beta diversity exhibited no significant differences between infants born through cesarean delivery and those born naturally who were fed with breast milk containing the α1-2 fucosylated HMOs, there were notable variations between the two groups. In infants born through CS, there was a lower abundance of Bacteroides and B. longum and a higher abundance of Akkermansia and Kluyvera in comparison with those born naturally. Furthermore, it was observed that infants born through CS to mothers with a positive secretory phenotype (producing α1-2 fucosylates) had a higher abundance of Akkermansia than those born through natural childbirth. Akkermansia has been touted as a possible candidate for improving gut health and managing conditions such as obesity and metabolic diseases [94]. The presence of this bacterium has been associated with improved body weight management, improving insulin sensitivity, and reducing inflammation [95]. Its beneficial nature has led to Akkermansia being considered as a possible candidate for use as a probiotic in promoting gut health. However, it is important to note that research on this front is still ongoing [96].

The study conducted by C. Mei Chien et al. [86] successfully addressed the intriguing issue of differences in microbiota between infants born through CS and those born naturally. Consistent with previous research, infants born through CS tend to experience delayed colonization by beneficial bifidobacteria are critical for immune system development and long-term health. The supplementation of a synbiotic mixture comprising short-chain galacto-oligosaccharides (scGOS), long-chain fructo-oligosaccharides (lcFOS), and Bifidobacterium breve M-16V was found to mitigate this discrepancy. This result is noteworthy as it suggests that a targeted dietary approach can help restore the GM of cesarean-born infants, bringing it closer to the composition found in naturally born infants. The reduced presence of Enterobacteriaceae, which are typically associated with pathological conditions, in infants treated with the synbiotic blend offers further evidence of the effectiveness of this strategy in fostering a more health-promoting gut environment. These findings offer novel insights into how targeted dietary modifications can positively influence the microbiota and potentially reduce the risks associated with cesarean deliveries. However, it is imperative to continue to explore the long-term effects of these modifications on the health and immunity of cesarean-born infants.

The colonization of an infant’s gut by microbiota during birth is a critical process, with the first months of life being pivotal to the establishment of the GM and immune system maturation. Disturbances in GM can lead to various metabolic and allergic diseases, including obesity, diabetes, and Crohn’s disease [97]. Prenatal and postnatal factors such as delivery mode, feeding pattern, and antibiotic usage influence the colonization of intestinal microorganisms. CS delivery, in particular, can disrupt the balance of intestinal flora with potential long-term health effects. Human milk is recognized as the gold standard for infant nutrition due to its prebiotic and probiotic components, particularly beneficial for the growth of bifidobacteria. In cases where breastfeeding is not feasible, infant formula should aim to support the development of the intestinal ecosystem.

This study by Ilias Lagkouvardos et al. [87] explored the effects of a synbiotic intervention formula (IF) enriched with L. fermentum CECT5716 and galacto-oligosaccharides (GOS) on fecal microbiota in infants using 16S rRNA gene amplicon sequencing and the measurement of milieu parameters. The results showed that the synbiotic intervention formula, when introduced during the early months of life, led to changes in the infant’s GM, increasing the relative abundance of bifidobacteria and reducing the richness and pH levels, thereby resembling some characteristics of breastfed infants.

Moreover, the impact of the intervention was dependent on the natural microbiota profiles of the infants, highlighting the individualized nature of gut microbiomes. These findings underscore the potential of synbiotic interventions to influence GM composition and milieu parameters during early life, with potential implications for disease prevention and the promotion of infant health, especially in cases of CS births where microbiota development may differ.

Joanna Hurkala, et al. [72] confirmed that infants born in the hospital by CS are virtually free of Lactobacillus and Bifidobacteria in their GM until days 5 and 6 after delivery, as these bacteria are virtually indistinguishable (below 2 log/g) in control infants. On the other hand, bacteria considered potential pathogens were present in both control and intervention infants. This observation confirmed previous findings that infants born through CS in hospitals are rapidly colonized by bacteria derived from the hospital environment, although it cannot be ruled out that a proportion of these bacteria (particularly coagulase-negative staphylococci) are transferred from the mother’s skin. One month after delivery, colonization with potential pathogens was more pronounced, which could reflect the natural process of acquiring bacteria from the environment, as Gram-negative bacteria, coagulase-negative staphylococci, and enterococci constituted the majority of this population. In conclusion, the study found that providing newborns delivered through CS with a combination of *Lactobacillus rhamnosus* and *Bifidobacterium brevis* immediately after birth results in increased populations of lactobacilli and bifidobacteria in their gastrointestinal tract, thus simulating the typical colonization found in newborns. born with natural birth.

The study by Wenqing Yang et al. [88] revealed that delivery mode significantly influenced neonatal GM composition. While there were no differences in microbial diversity between cesarean and vaginally born infants on the third day, significant differences emerged on the seventh and twenty-eighth days. These findings suggest that the delivery mode can influence neonatal GM.

Furthermore, probiotic supplementation showed varying effects on the composition of GM. After three days of probiotic supplementation, Bifidobacterium abundance significantly increased in cesarean-born infants. Similarly, Lactobacillus abundance was positively impacted by probiotics in the early neonatal period. Notably, low-dose probiotic supplementation appeared to have a more pronounced effect. Analysis of the Clusters of Orthologous Groups of proteins (COG) functions in GM indicated that probiotics could impact the microbiota’s function. The relative abundance of COGs related to basal metabolism, nucleotide metabolism, transport, and defense mechanisms appeared to change due to probiotic supplementation.

In conclusion, this study suggests that supplementing probiotics to cesarean-born neonates can partially restore changes in fecal microbiota composition. Delivery mode plays a significant role in determining neonatal GM composition.

The first 1000 days of a child’s life represent a critical period for health and development. During this time, the composition of the microbiome plays a vital role, and any disruptions in its establishment can lead to non-communicable diseases later in life. Factors such as exposure to antibiotics, CS birth, and immune and metabolic health have been linked to conditions like asthma, eczema, obesity, and type 2 diabetes. A recent study has even found a connection between CS births and an increased risk of infection-related hospitalizations in early childhood. Key microorganisms known as keystone colonizers, including Bifidobacterium and Bacteroides, are crucial for immune programming and maintaining a healthy symbiosis with the human host. One study suggested a method of swabbing infants born through CS with vaginal secretions to partially restore the lack of maternal microbiota transmission. However, concerns about infection risk have been raised.

In this study by Lay et al. [89], a specific synbiotic intervention, consisting of scGOS/lcFOS and Bifidobacterium breve M-16 V, was administered to infants born through elective CS. This intervention aimed to restore delayed colonization and potentially reduce the incidence of conditions like eczema and atopic dermatitis. The research involved sequencing and metabolomic analysis of fecal samples from various groups of infants, including those born through CS and those born vaginally. The results showed that the mode of delivery had a significant impact on the development of the infant’s GM. CS-born infants exhibited delayed colonization by keystone colonizers compared with vaginally born infants alongside an increased abundance of Enterobacteriaceae. Further analysis revealed that the synbiotic intervention effectively modulated the GM of CS-born infants, leading to a microbial environment characterized by strict anaerobes, similar to that of vaginally born infants. Bifidobacterium played a central role in this modulation, producing organic acids like acetic acid that contributed to the anaerobic environment and improved epithelial barrier function.

The study also highlighted the importance of early-life microbiome establishment in maintaining gut health. The presence of Bifidobacterium in the first days of life appeared to be critical in modulating the gut’s redox and acidity, providing colonization resistance and programming the immune system. Delayed colonization by Bifidobacterium was associated with an increased risk of pediatric allergies.

In conclusion, this research sheds light on the significance of early-life microbiome modulation in CS-born infants and its potential impact on long-term health. The synbiotic intervention showed promise in restoring a compromised microbiome, emphasizing the need for further investigation into its long-term effects on child health.

On the other hand, in the comprehensive study by Joanne E Sordillo et al. [69], the intricate relationship between an infant’s GM and various prenatal and early life factors was delved into, striving to shed light on their potential implications for immune system modulation and the incidence of asthma and allergies in childhood.

The results unveiled several paramount associations. First and foremost, the mode of delivery exhibited a profound influence on the infant gut microbiome. Infants born through CS displayed higher microbial diversity, a finding somewhat contrary to previous reports. However, further exploration revealed that CS-born infants exhibited an enrichment of proteobacteria, notably Klebsiella and Enterobacteriaceae, while levels of Bacteroides were reduced. This shift may have significant implications for immune stimulation and microbial function within the gut.

Breastfeeding emerged as another crucial determinant of an infant’s GM. Exclusive breastfeeding was linked to a decrease in overall diversity and a reduction in specific genera of Clostridiales such as Clostridium, Ruminococcus, Coprococcus, and Eubacterium. These findings underscore the potential role of breast milk components, such as prebiotic oligosaccharides, in shaping the gut microbiome.

Additionally, they identified racial and ethnic disparities in infants’ GMs. Caucasian infants exhibited lower microbial diversity but higher levels of Bacteroides, while African American infants displayed higher levels of Megasphaera and Lactococcus abundance. These disparities could contribute to variations in asthma incidence among different racial groups.

Furthermore, cord blood vitamin D levels were associated with specific alterations in the infant gut microbiome. Higher vitamin D levels were linked to increased Lachnospiraceae/U, Clostridiales, and Lachnobacterium as well as decreased Lactococcus.

In conclusion, this research, which was conducted within one of the most extensive and ethnically diverse infant study populations, provided valuable insights into the factors shaping the infant gut microbiome. These findings underscore the significance of delivery mode, breastfeeding practices, race, and vitamin D levels in influencing the composition of the GM during this critical developmental period. Further investigations are needed to unravel the precise mechanisms through which these microbiome alterations may impact immune system function and the risk of asthma and allergic diseases in childhood. Longitudinal studies tracking microbiome changes over time and elucidating their relationship with health outcomes will be essential in advancing our understanding of the microbiome’s role in these conditions.

The study by Li et al. [90] investigated the efficacy of *Lactobacillus paracasei* N1115 (Lp N1115) as a probiotic in improving gut microbial composition and immunomodulation among Chinese infants and children born through CS.

As far as gut microbial composition is concerned, Lp N1115 intervention increased the relative abundance of Lactobacillus in the experimental group compared with the control group, particularly at week 4 (*p* = 0.019). In addition, a trend toward a higher detection rate of Lactobacillus was observed in the experimental group (*p* = 0.039).

Furthermore positive correlations were found between Lactobacillus abundance and sIgA levels in the feces of infants as well as a negative correlation between fecal pH and Lactobacillus abundance in 6- to 12-month-old infants.

In conclusion, their study shows that Lp N1115 supplementation in Chinese infants and children born through CS has several beneficial effects, including maintaining fecal pH levels, reducing stress, increasing fecal sIgA levels, and promoting Lactobacillus proliferation. These results suggest the potential of Lp N1115 as a probiotic to support healthy gut development in this specific population. Further research is needed to explore its long-term impact and optimal intervention strategies.

Moreover, it is essential to consider the role of antibiotics in shaping the infant gut microbiota. The administration of antibiotics, particularly during and after CS deliveries, has been identified as a significant factor influencing microbial colonization [98]. A study conducted by Thomas Dierikx et al. [91] delved into the impact of maternal antibiotic administration on the microbial colonization process in infants born through CS up to three years of age. Their findings revealed that CS delivery profoundly affects early-life microbiome development, with differences in microbial diversity and composition observed in both CS groups compared with vaginally born infants. Notably, maternal antibiotic administration prior to CS did not appear to exacerbate this colonization impairment, suggesting that antenatal antibiotic exposure in CS-born infants does not result in a secondary hit on the already compromised microbiome. These results have significant implications, especially given the increasing rates of CS worldwide. They shed light on the microbiota-related consequences of maternal antibiotic prophylaxis resulting from CS and provide valuable insights into the long-term health outcomes of CS-born infants. Further research with larger sample sizes ought to validate these findings and help allay concerns regarding the impact of antenatal antibiotics on the developing infant microbiome and long-term health.

## 4. Conclusions

In conclusion, the various studies discussed in this article shed light on the significant impact of delivery mode, particularly CS, on the early GM in infants and its potential implications for long-term health. These studies collectively emphasize the importance of understanding and intervening in this critical period of microbiome development to reduce the risks associated with disturbances in the GM. The key findings from these studies can be summarized as follows:

1. CS vs. vaginal birth: Infants born through CS tend to exhibit significant differences in their GM compared with those born naturally. There is a consistent reduction in beneficial bacteria, including Bifidobacterium and Bacteroides, in CS-born infants.
2. Probiotic and synbiotic interventions: Some studies suggest that probiotic and synbiotic supplements can help mitigate the negative effects of CS deliveries on an infant’s GM. These interventions promote the colonization of beneficial bacteria and a more balanced microbiome that is crucial for the infant’s long-term health.
3. Vaginal seeding: The efficacy of vaginal seeding remains a topic of debate. While some studies did not find significant microbiota changes in CS-born infants after vaginal seeding, concerns about infection risks have been raised. Alternative strategies, like probiotics and delayed antibiotic administration, may offer safer options.
4. Breastfeeding: The composition of human milk, particularly the presence of human milk oligosaccharides (HMOs), can influence the GM in infants. It is essential for the growth of beneficial bifidobacteria.
5. Prenatal and early life factors: Various factors, including delivery mode, feeding patterns, and antibiotic usage, influence the colonization of an infant’s GM. Identifying these factors helps us to understand how they impact the infant’s long-term health.
6. Race and ethnicity: Some studies have shown racial and ethnic disparities in infants’ GMs, which can have implications for variations in disease incidence.
7. Maternal antibiotic use: Maternal antibiotic use during CS delivery does not appear to exacerbate colonization disturbances in infants, indicating that antenatal antibiotic exposure may not result in a secondary hit on the infant’s already compromised microbiome.
8. Long-term health implications: Early-life microbiome modulation and interventions, especially in CS-born infants, may have far-reaching implications for long-term health outcomes, including reducing the risk of diseases like asthma, allergies, obesity, and diabetes.

In conclusion, understanding and intervening in the establishment of an infant’s GM during the first 1000 days of life is critical for preventing various health issues in later life. Further research, with larger sample sizes and longer follow-up periods, is needed to explore the long-term effects of these interventions and to clarify the intricate relationships between maternal, prenatal, and postnatal factors and an infant’s GM. This body of research paves the way for strategies to promote healthy microbiome development and reduce the risks associated with CS births and other factors that can disrupt the infant’s microbial ecosystem.

## Figures and Tables

**Table 1:** Database search indicators.

Articles screening strategy	KEYWORDS: A: cesarean delivery; B: infant gut microbiota; C: infant oral microbiota.
	Boolean Indicators: A AND (B OR C)
	Timespan: 2013–2023
	Electronic databases: Pubmed; Scopus; WOS

**Table 2:** Descriptive summary of item selection.

Authors (Year)	Study Design	Number of Patients	Materials and Methods	Outcomes
K. Korpela et al., 2018 [83]	Randomized clinical trial	428 infants	The study conducted an analysis of the GM composition in 428 infants at 3 months of age using fecal samples and 16S rRNA gene amplicon sequencing. Mothers were randomized into control and treatment groups during pregnancy, with the treatment group receiving a mixture of specific bacterial strains. Infants continued to receive these capsules after birth, and fecal samples were collected for analysis. Information on birth mode, breastfeeding, formula feeding, and antibiotic use was obtained through questionnaires.	Newborns’ microbiota composition was significantly influenced by probiotic supplementation; breastfed newborns had higher levels of bifidobacteria and lower levels of proteobacteria and clostridia. Probiotics reversed or lessened the effects of antibiotic usage and birth mode, which were linked to altered microbiota in the placebo group.
B. C. Wilson et al., 2021 [84]	Randomized clinical trial	47 babies	Healthy babies born through cesarean delivery were randomly assigned to receive either sterile water (CS-placebo, *n* = 13) or a 3 mL solution of maternal vaginal microorganisms (CSseeded, *n* = 12). Neonatal infants born vaginally (VB, *n* = 22) served as the reference control. Clinical evaluations were performed within the first two hours after birth as well as at one and three months of age. Shotgun metagenomic sequencing was performed on maternal vaginal extracts and infant stool samples from CS women. The composition of the GM at one month of age was the main result. The functional potential of the gut microbiome, maternal strain engraftment, anthropometry, body composition, and adverse events were all secondary outcomes.	The results showed that vaginal seeding had no discernible effect on the microbiome development of CS-born infants, particularly with regard to Bacteroide colonization, regardless of the dosing technique. Therefore, for infants born through CS, maternal fecal microbiota transplantation (FMT) might be a more successful strategy. Additionally, it was discovered that intrapartum antibiotic prophylaxis (IAP), which is frequently used during CS, decreased the exposure of newborns to maternal microbes and might have a deleterious impact on the survivability of transplanted microbes. In summary, this pilot study suggests that the oral administration of maternal vaginal microbiota did not significantly affect the early gut microbiome of CS-born infants, questioning the utility of this procedure in reducing disease risk.
K. M. Tonon et al., 2021 [85]	Cross-sectional study	48 infants	This study involved a subset of mother–infant pairs participating in a cross-sectional observational study aimed at identifying factors associated with human milk oligosaccharide (HMO) concentrations. The participants included healthy full-term singleton infants who were exclusively breastfed and had not received antibiotics, probiotics, water, or any other food besides human milk. Human milk and infant fecal samples were collected at one month postpartum and processed for analysis. Human milk samples were stored at −20 °C for HMOs analysis. Infant feces were collected from disposable diapers, preserved in an ASL buffer, and stored at −20 °C for DNA extraction. The study involved analyzing the fecal microbiota composition through 16S rRNA gene sequencing, and the main bacterial genera and species were quantified using a qPCR with specific primers. Standard curves for quantification were created using reference gene fragments, and results were expressed as bacterial units per gram of feces (U/g of feces). The detection limit for all organisms was 1 cell/g.	The researchers found that infants born through cesarean had lower levels of Bacteroides, less B. longum, and higher levels of Akkermansia as well as Kluyvera in their GM. Despite these differences, the overall composition of the microbiota did not differ significantly between infants born through cesarean and those born vaginally, provided they were breastfed by secretory mothers. In addition, the study noted an increased presence of Verrucomicrobia and Akkermansia, mainly in CSe+ infants. Akkermansia is a bacterium involved in immune regulation and promotion of the intestinal barrier function, which also is associated with lower risks of obesity and allergies in infants. Another distinctive observation was the higher prevalence of proteobacteria, particularly Serratia and Kluyvera, in this group. This differed from previous studies, probably because of socioeconomic differences between the populations.
C. Mei Chien et al., 2017 [86]	Randomized Clinical Trial	152 babies	Infants were given either a non-hydrolyzed cow’s milk-based formula (control formula), a prebiotic formula supplemented with 0.8 g/100 mL of scGOS/lCFOS, or a synbiotic formula supplemented with B. breve M-16V (Morinaga Milk Industry Co. Ltd.) at a dose of 7.5 10^8^ cfu/100 mL. As a reference group, vaginally delivered infants were included. From birth (1–3 days at the latest) until 16 weeks of age (the intervention phase), and study formulae were given. At day 3, day day 5, week 2, week 4, week 8, week 12, and week 22, stool samples were taken.	This study showed that supplementation with scGOS/lcFOS and B. breve M-16V contributes to the early colonization of bifidobacteria in infants born through CS, reproducing the physiological conditions of the intestinal microbiota observed in vaginally born infants. Positive effects have also been observed in terms of the reduction in adverse events such as skin disorders, particularly eczema/atopic dermatitis.
Ilias Lagkouvardos, 2022 [87]	Randomized Clinical Trial	540 infants	- Synbiotic intervention formula (IF) containing Limosilactobacillus fermentum CECT5716 and galacto-oligosaccharides. - Measurement of metabolites (e.g., short-chain fatty acids) and milieu parameters (e.g., pH, humidity, and IgA) in stool samples.	- Fecal microbiota analysis through 16S rRNA amplicon sequencing at 4, 12, and 24 months of age. - Significant changes in microbiota profiles with age. - Closer overall phylogenetic profiles of infants receiving IF to those fed with human milk at month 4. - Association of these microbiota states with higher prevalence of infants born through CS.
Wenqing Yang et al., 2021 [88]	Observational Cohort Study	26 neonates	- Neonates divided into four groups as follows: VD (natural delivery neonates, *n* = 3), CD (cesarean-born neonates, *n* = 9), CDL (cesarean-born neonates supplemented with a probiotic at a lower dosage, *n* = 7), CDH (cesarean-born neonates supplemented with a probiotic at a higher dosage, *n* = 7) - Sequencing of the V3–V4 region of the 16S ribosomal ribonucleic acid gene through next-generation sequencing technology	- α-diversity of intestinal microbiota significantly lower in cesarean-born neonates on the 28th day compared with naturally delivered neonates (*p* = 0.005). - Abundances of Lactobacillus and Bifidobacterium significantly increased from the 3rd day of probiotic supplementation. - Impact of probiotic supplementation on the diversity and function of GM.
Christophe Lay et al., 2021 [89]	Double-blind randomized controlled study	153 infants	Newborns’ microbiota compositions were significantly influenced by probiotic supplementation; breastfed newborns had higher levels of bifidobacteria and lower levels of proteobacteria and clostridia. Probiotics reversed or lessened the effects of antibiotic usage and birth mode, which were linked to altered microbiota in the placebo group.	Babies delivered vaginally had an environment in their guts that was hypoxic and acidic, with a higher concentration of stringent anaerobes (Bifidobacteriaceae). Enterobacteriaceae enrichment is a sign of a damaged microbiome in infants born after cesarean delivery.
Joanne E Sordillo et al., 2017 [69]	Clinical trial	333 infants	Microbial diversity was calculated using the Shannon index, and 16S rRNA gene sequencing was employed for infants’ stool samples.	White race/ethnicity was associated with lower diversity but higher Bacteroidetes coabundance scores. CS birth was associated with higher diversity but decreased Bacteroidetes coabundance scores. Infants born through CS had higher firmicutes scores. Infants that were breastfed showed reduced levels of Clostridiales. Vitamin D in cord blood is associated with a rise in Lactococcus but a decrease in lactobacteria.
Pin Li et al., 2023 [90]	Randomized, placebo-controlled trial	109 infants	Saliva and stool samples collected at weeks 0, 4, 8, and 12 from infants aged 6–24 months born through CS.	- Fecal pH increase in the control group (*p* = 0.003). - No change in fecal pH in the experimental group. - Salivary cortisol decrease in the experimental group (*p* = 0.023). - Little change in cortisol in the control group. - No obvious effect on fecal calprotectin and saliva sIgA. - Enhancement of Lactobacillus content.
Thomas Dierikx et al., 2022 [91]	Randomized controlled trial	CS group (*n* = 40); vaginal group (*n* = 23)	Microbiota analyzed through 16S rRNA gene sequencing and whole-metagenome shotgun sequencing. Data collected at 1, 7, and 28 days after birth and at 3 years.	- Microbial diversity and composition compared between CS and vaginal groups in the first month of life. - Abundance changes in specific bacterial genera noted. - Comparison of microbiome at 3 years of age. - Confirmation of CS delivery’s impact on microbiome colonization.
Yang Liu MD et al., 2023 [10]	Randomized clinical trial	A total of 120 pregnant women were divided into two groups as follows: a “vaginal seeding” group (*n* = 60) and a control group (*n* = 60).	This randomized controlled trial was conducted at the Liuyang Maternal and Child Health Care Hospital in China to investigate differences in GM between infants born through cesarean delivery and those born through natural childbirth. In the “vaginal seeding” group, sterile gauze soaked in sterile saline solution was inserted into the maternal vagina one hour before delivery and then used to gently swab the infant’s body after birth. In contrast, the control group received standard care.	The results of the study revealed that there were no significant differences in the GM between the two groups of infants. The analyses found that changes in gut bacterial composition were similar in both the “vaginal seeding” group and the control group. In addition, no significant differences in BMI (body mass index) or allergy risks were found between the two groups during the infants’ first 2 years of life.
Joanna Hurkala et al., 2020 [72]	Randomized clinical trial	The recruited infants (148) were divided into two groups as follows: the intervention group (71) and the control group (77).	Newborns were divided into two groups as follows: one group received a probiotic product with specific strains of Bifidobacterium and Lactobacillus shortly after birth, while the other group served as the control. The study aimed to investigate the impact of probiotic supplementation on the early GM of newborns born through CS. Stool samples were collected on days 5 or 6 after birth and again after one month. These samples were analyzed to assess the presence and quantity of bacterial genera and species, including beneficial ones like Lactobacillus and Bifidobacterium as well as potentially harmful bacteria.	The intervention group showed a significant increase in Lactobacillus and bifidobacteria levels in their fecal samples compared with the control group. Lactobacillus levels were high, while bifidobacteria levels were higher, indicating the effectiveness of probiotic supplementation in infants.

## Data Availability

Not applicable.

## Abbreviations

CS	Caesarean section
CD	Cesarean-born neonates
CDL	Cesarean-born neonates supplemented with a probiotic at a lower dosage
CDH	Cesarean-born neonates supplemented with a probiotic at a higher dosage
COG	Clusters of orthologous groups of proteins
FMT	Fecal microbiota transplantation
GM	Gut microbiota
GOS	Galacto-oligosaccharides
sIgA	Fecal secreted immunoglobulin A
HMOs	Human milk oligosaccharides
IF	Intervention formula
Lp N1115	Lactobacillus paracasei N1115
MS	Mass spectrometry
scGOS/lcFOS	Short-chain galactooligosaccharides and long-chain fructooligosaccharides
PCR	Polymerase chain reaction
RCT	Randomized clinical trial
VD	Natural delivery neonates
VDAART	Vitamin D antenatal asthma reduction trial
